# An Unusual Case of CMV/EBV Ventriculoencephalitis with Evolution to Primary Central Nervous System Lymphoma in HIV-Positive Patient

**DOI:** 10.1155/2018/7683797

**Published:** 2018-07-17

**Authors:** Gisela Borges, Diana Neves, Inês Pintado Maury, Aida Pereira, Maria de Jesus Silva

**Affiliations:** ^1^Internal Medicine Department, Hospital Pulido Valente, Lisbon, Portugal; ^2^Infectious Diseases Department, Hospital Santa Maria, Lisbon, Portugal

## Abstract

Epstein–Barr virus (EBV) is a well-known cause of different types of malignancies particularly Burkitt's lymphoma, nasopharyngeal carcinoma, Hodgkin's lymphomas, and non-Hodgkin's lymphomas including primary central nervous system lymphoma (PCNSL). A higher tendency of malignant transformation associated with EBV has been noticed in immunocompromised patients, such as human immunodeficiency virus (HIV) infected patients. The rapid and effective immune reconstitution is crucial to prevent PCNSL in HIV-positive patients. We present a clinical case of a young patient diagnosed with HIV infection and medicated with antiretroviral therapy (ART) with poor immunological recovery. After two weeks, he developed ventriculoencephalitis, observed in the cranial magnetic resonance imaging (MRI), caused by cytomegalovirus (CMV) and EBV, both with high serum viral load, rapidly evolving to PCNSL. With this unusual clinical case, the authors want to draw attention to the importance of rapid immunological reconstitution in preventing the progression of EBV infection to PCNSL, as well as encouraging the confirmation of the usefulness of early combination of chemotherapy and antiviral therapy, in order to reach a more effective treatment of this herpesvirus infection and associated malignancies.

## 1. Introduction

HIV infected patients, especially with low CD4+ T cell count, are more susceptible to severe central nervous system (CNS) infections. Several studies have shown that EBV DNA detection in cerebrospinal fluid (CSF) is a good PCNSL marker in this group of patients [1]. Nonetheless, focal brain lesion stereotactic biopsy is the gold standard procedure to establish the final diagnosis. A prompt HIV infection diagnosis and a highly active antiretroviral therapy (HAART) initiation are essential measures to achieve an immunological recovery and consequently prevent EBV infection and its progression to PCNSL [2]. The association between antiviral therapies with specific viral acting chemotherapy (rituximab) may be a more effective therapy against EBV replication and associated PCNSL. The prognosis of patients with EBV associated PCNSL is poor. The average life expectancy varies between two and twelve months after diagnosis [3].

## 2. Case Report

**Figure 1:**
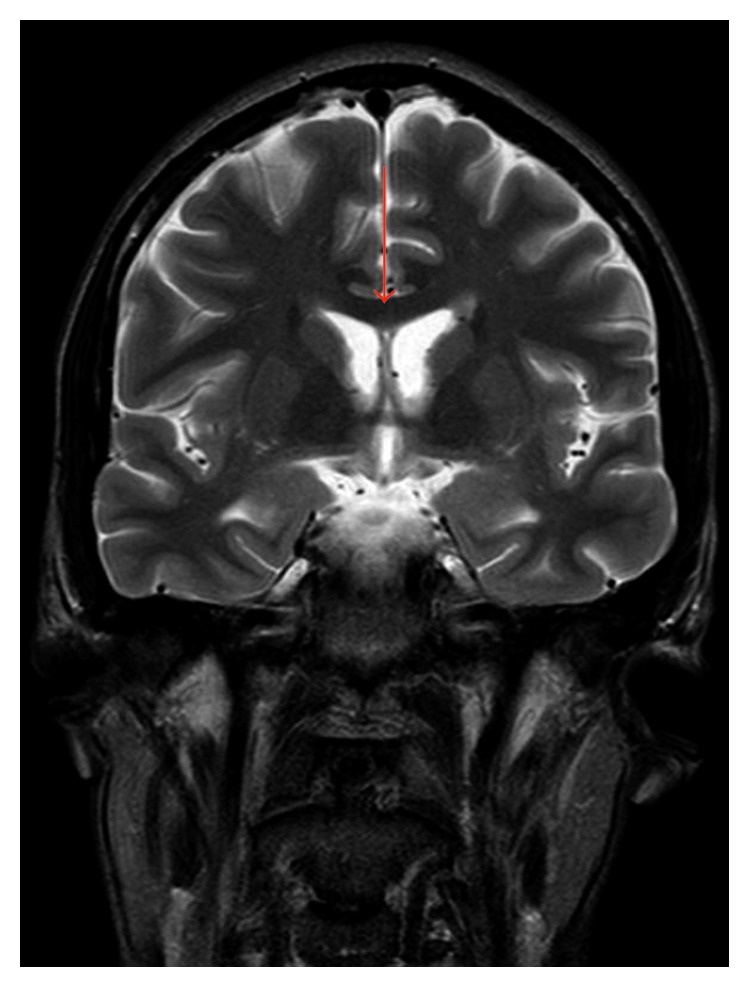
Ventricular hypersignal in cranial MRI (FLAIR), suggestive of ventriculitis.

We present a clinical case of a 31-year-old man diagnosed with HIV-1 infection, with CD4 T cell count of 35 cells/mm^3^ (4%) and HIV RNA 305349 copies/mL (log^10^ 5.48) having initiated ART with abacavir/lamivudine and nevirapine. Around two weeks after starting ART, the patient is admitted due to a sudden cognitive impairment (anhedonia and memory loss) with progression to gait change and imbalance. The cranial computerized tomography (CT) scan showed no lesions but the cranial MRI revealed ventriculoencephalitis (Figure 1).

The cerebrospinal fluid (CSF) had 38 nucleated cells/mm_3_, 175 mg/dL proteins and 37 mg/dL glucose (glycaemia 82 mg/dL). The CSF CMV and EBV viral load were 189000 (log^10^ 5.28) and 799 (log^10^ 2.90) copies/mL with negative CSF neurotropic microorganism serologies and molecular identification (HSV 1/2, VZV, *Cryptococcus*, *Brucella*, *Treponema pallidum*, *Borrelia burgdor*feri, JC virus, *Mycobacterium tuberculosis*, and *Toxoplasma gondii*). The final considered diagnostic was mainly CMV-related ventriculoencephalitis and ganciclovir was started.

Nevertheless, the patient started left conjugate horizontal gaze palsy with abducting horizontal saccadic (or jerk-type) nystagmus of the right eye as well as a slight anisocoria with left eye miosis. These changes were enclosed in the one-and-a-half syndrome and left-sided Horner's syndrome. The patient also presented a grade II-III paresis of the right lower limb. The cranial CT scan (performed fifteen days later) revealed a dubious right linear protuberancial hypodensity without signs of intracranial hypertension.

Cranial MRI was repeated one month later revealing improvement of the ventriculitis signs but a larger hippocampus and left mesial temporal region involvement with a discrete increase of the lateral ventricles dimensions.

Due to these clinical and imagiological changes and because we could not exclude tuberculosis infection, classic first-line tuberculostatic therapy was empirically started (stopped after excluding this infection) and foscarnet was added to ganciclovir (until a negative CMV viral load was achieved). At this point, the hypotheses of limbic encephalitis, epileptic activity or paraneoplastic encephalitis could not be excluded. The lumbar puncture was repeated and CSF antineuronal antibodies, HHV-8, and other neurotropic microorganisms were negative.

**Figure 2:**
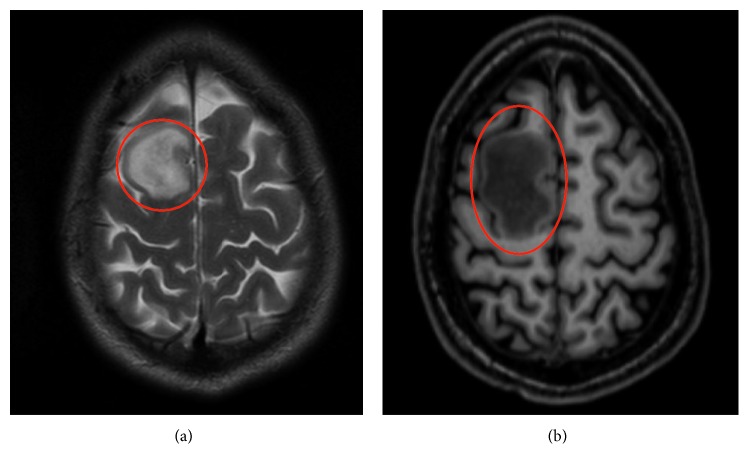
Cranial MRI in T2 and T1, respectively—frontal lesion corresponding to PCNSL.

The electroencephalogram (EEG) showed frontal and frontotemporal bilateral activity with occasional periodic discharges, independent of nonabrupt three-phase bifrontal lesions. Anticonvulsant therapy with levetiracetam and topiramate was started. One month later, the cranial MRI was repeated and a new frontal lesion was detected. The body CT scan was normal. The imagiological reevaluation showed a slight improvement of the frontal lesion with new cerebellum lesions. The suspicion of lymphoproliferative disease was meaningful. Cranial MRI with diffusion and perfusion study with spectroscopy was performed and revealed very suggestive images of CNS lymphoma (Figure 2). For definitive diagnosis, a stereotactic brain biopsy was done. The histological study revealed a polymorphic cellular infiltrate with histiocytes, CD3+ T-lymphocytes, and CD 20+ B-lymphocytes (mixture of numerous small cells and large activated cells with positive hybridization results for EBV-encoded RNA (EBER)). The histology was suggestive of polymorphic lymphoproliferative EBV-positive disease. The molecular studies were not done because the sample was insufficient to perform the clonality analysis (CSF EBV viral load was 12405 copies/mL; CSF HIV and CMV viral load were both undetectable).

At this point, chemotherapy with rituximab was started and radiotherapy was scheduled. Nevertheless, the patient developed a sudden neurological worsening with a generalized tonic-clonic seizure, refractory to the instituted measures, culminating in death.

## 3. Discussion

The incidence of PCNSL in HIV infected patients is between 2 and 6% (1000 times higher than in general population) and more than 10% in postmortem diagnosis. Risk factors for the development of non-Hodgkin's lymphoma in these patients are low CD4+ T cell count, elevated HIV serum viral load, and chronic hepatitis B and C infection in patients receiving ART [1]. The reported clinical case is about a patient who developed CMV/EBV ventriculoencephalitis that, despite the virological response to antiviral therapy with ganciclovir and foscarnet, evolved to EBV-driven B cell lymphoproliferative disorder that is considered a “grey disorder” (neither benign nor malignant) that usually evolves to PCNSL [4].

Concerning HIV infection, he had a low immunological status on ART with two of the previously mentioned risk factors: CD4+ T cell count 35 cells/mm^3^ (4%) and HIV 305349 RNA copies/mL (log^10^ 5.48). It is well known that HAART is able to improve the immune system, being one of the best measures to prevent the EBV infection progression to PCNSL. In HIV infected patients, CMV is usually the most frequent agent associated with CNS infections; nevertheless, the detection of EBV, herpesvirus 6 (HHV-6), and mixed infections may also occur [2]. HIV associated cytomegalovirus (CMV) encephalitis is one of several central and peripheral nervous system infections seen in late-stage disease. Neurologic manifestations of CMV infection include encephalitis, ventriculitis, myelitis, retinitis, radiculoganglionitis, and peripheral neuropathies. These infections usually occur in patients with severe immunodeficiency: CD4+ T cell count typically is lower than 50 cells/mm^3^, but even in these patients, viral ventriculoencephalitis cases are rare, which makes this case more interesting. Prior to the development of HAART, 2% of HIV infected patients with CD4+ T cell count less than 50 cells/mm^3^ developed CMV neurologic disease [5]. If left untreated, HIV associated CMV encephalitis typically progresses to death in days to weeks. Many studies have shown that EBV DNA detection in CSF is a good lymphoid proliferation and PCNSL diagnostic marker in HIV-positive patients [1].

EBV expresses several genes (latent genes) that participate in the pathogenesis of specific neoplasms, including PCNSL. It has been documented that EBV can be detected in more than 50–60% of lymphoma cases in HIV infected patients [6]. PCNSL accounts for up to 15% of non-Hodgkin's lymphomas in HIV infected patients compared to only 1% in general population. Approximately 20 to 30% of the central nervous system lesions in patients with acquired immunodeficiency syndrome eventually show as PCNSL (approximately half present multiple lesions), with toxoplasmosis and progressive multifocal leukoencephalopathy being the remaining cases. A high level of clinical suspicion is crucial for prompt diagnose and therapeutic management of these patients. Nowadays, there are several suggested therapies for EBV associated PCNSL; however, guidelines have not yet been established. According to the reviewed literature, antiviral therapy efficacy is higher when the virus is in its lithic phase; therefore, one of the new approaches includes induction of this phase by chemotherapy, followed by treatment with antiviral. However, its efficacy has not yet been well studied. On the other hand, monoclonal antibodies, such as anti-CD20 (rituximab), have shown promising results [7]. Although our patient initiated rituximab immediately after the final diagnosis, he had a neurological deterioration culminating in death. This final denouement most likely happened due to the difficult process of reaching the final diagnosis of a disease that itself has a poor prognosis.
